# Testing the Effects of COVID-19 Confinement in Spanish Children: The Role of Parents’ Distress, Emotional Problems and Specific Parenting

**DOI:** 10.3390/ijerph17196975

**Published:** 2020-09-24

**Authors:** Estrella Romero, Laura López-Romero, Beatriz Domínguez-Álvarez, Paula Villar, Jose Antonio Gómez-Fraguela

**Affiliations:** Department of Clinical Psychology and Psychobiology, Universidade de Santiago de Compostela, 15782 Santiago de Compostela, Spain; laura.lopez.romero@usc.es (L.L.-R.); beatrizdominguez.alvarez@usc.es (B.D.-Á.); paula.villar@usc.es (P.V.); xa.gomez.fraguela@usc.es (J.A.G.-F.)

**Keywords:** COVID-19, confinement, children adjustment, family adjustment, specific parenting, psychological impact

## Abstract

The present study aimed to examine the effects of the Spanish confinement derived from the COVID-19 crisis on children and their families, accounting for child’s age. A range of child negative (e.g., conduct problems) and positive outcomes (e.g., routine maintenance) were examined, along with a set of parent-related variables, including resilience, perceived distress, emotional problems, parenting distress and specific parenting practices (e.g., structured or avoidant parenting), which were modeled through path analysis to better understand child adjustment. Data were collected in April 2020, with information for the present study provided by 940 (89.6%) mothers, 102 (9.7%) fathers and 7 (0.7%) different caregivers, who informed on 1049 Spanish children (50.4% girls) aged 3 to 12 years (M_age_ = 7.29; SD = 2.39). The results suggested that, according to parents’ information, most children did not show important changes in behavior, although some increasing rates were observed for both negative and positive outcomes. Child adjustment was influenced by a chain of effects, derived from parents’ perceived distress and emotional response to the COVID-19 crisis, via parenting distress and specific parenting practices. While parenting distress in particular triggered child negative outcomes, specific parenting practices were more closely related to child positive outcomes. These findings may help to better inform, for potential future outbreaks, effective guidelines and prevention programs aimed at promoting the child’s well-being in the family.

## 1. Introduction

The COVID-19 outbreak, rooted in a new strain of the coronavirus family, has caused a great impact, at many different levels, in our society. In December 2019, the disease started to spread in the city of Wuhan, China, with an enormous rate of contagion and fatalities that led the World Health Organization (WHO) to declare a public health emergency of international concern on 30 January 2020. A few weeks later, the disease had been detected in dozens of countries, with COVID-19 being declared a pandemic by the WHO on 11 March 2020. During the first months of the pandemic, Italy and Spain were, after China, the most affected countries, being the first regions—outside Asia—to suffer a severe disruption not only in health systems, but also in the population’s expectations, lifestyles and economic condition. During the spring of 2020, governments all over the world were forced to impose drastic measures to restrict people’s movements to curb the spread of the virus. Millions of people were required to stay home for weeks, with schools closed and non-essential business abruptly halted. Hence, in addition to the huge personal losses (31,425,0.29 confirmed cases and 967,164 deaths reported to the WHO by 23 September 2020), the pandemic caused an economic and social burden that is difficult to estimate.

Even considering that research on COVID-related effects is still in its infancy, the COVID-19 crisis has already shown an important psychological impact on individuals. A nationwide survey in China showed that almost 35% of the population experienced significant psychological distress, and a variety of psychological problems—i.e., anxiety and depression—were triggered by the current crisis [1]. In Western countries, evidence of emotional harm was also found during the acute period of the pandemic [2]. In this regard, the study and protection of mental health during the pandemic crisis emerges as one of the most important challenges for science, policy makers and administrations [3].

Many commonalities have been identified between pandemics and other previous disasters (e.g., natural disasters, war, terrorism), which also caused a great impact on communities, and an increased rate of unpredictability and fatalities [4]. Hence, the literature about disasters, stress and trauma is currently inspiring the study of the psychological effects of COVID-19 [5]. As observed in previous research about disasters [6], children can be particularly vulnerable to the COVID-19 effects, as they have more limited resources to understand and interpret the events associated with the pandemic. In addition, the restrictive lockdown imposed in many countries, including Spain, for many weeks, involved a sudden change in children’s routines, took them away from their social contacts at school and their leisure activities outdoors and, overall, subjected them to the stress of restructuring daily life. Accordingly, from the very beginning of the COVID-19 outbreak, calls have been made to pay close attention to the effects of the confinement in childhood [7].

### 1.1. Psychological Impact of Confinement in Children

The effects of the social distancing and isolation measures (i.e., confinement) in health-related disasters were analyzed in some previous research. In a retrospective study on the reactions to pandemic episodes, Sprang and Silman [4] found that about 30% of children could have experienced significant post-traumatic stress symptoms. In the context of the current COVID-19 crisis, the first results about the effects on children were reported in China [8], with rates of 17.2% and 22.6% for anxiety and depression, respectively, which reveals a higher ill-being level than observed in regular conditions. In Europe, Orgilés et al. [9] examined child behavior and emotional change perceived by parents during the confinement in Italy and Spain. Results showed that the effects of the confinement were stronger in Spain (probably due to the more restrictive lockdown, with children forced to be at home for more than 40 days). Overall, parents reported change in 86.7% of children, with difficulties in concentration as the most frequent symptom (reported by 76.6% of parents), followed by boredom, irritability, restlessness, nervousness, loneliness, uneasiness and worriedness (observed by more than 30% of parents).

Notwithstanding that the negative effects of the pandemic on the psychological well-being of children should be thoroughly analyzed, researchers should also focus on potential positive outcomes derived from this crisis. Previous research on risk and resilience in disasters showed that, in adverse situations, it might be possible to find positive patterns of adaptation, perceived benefits or even post-traumatic growth [10]. Positive changes in relation to disasters were described in adults [11] and, to a lesser extent, in children [12]. In previous outbreaks (e.g., SARS in 2003), it was reported that many people experienced a feeling of embeddedness in the community, prosocial behaviors toward friends and family, and better routines of self-care [13]. Similarly, in the current COVID-19 pandemic, a blossoming of solidarity and altruism was reported [14]. Nevertheless, is still unknown to what extent children exposed to health-related disasters experience similar positive consequences, encompassing, for instance, increased cooperation, social bonding, evolved conceptions about society or stronger connections to their immediate family through spending more time at home.

Expectedly, there might be differences in the way that children react to the confinement. While it is likely that most children show negative emotions and behaviors, it could also be expected that some children experience positive outcomes. The potential heterogeneity in child outcomes needs to be further explained and, in search of possible determinants, family constitutes a paramount setting [15]. As shown by the evidence on positive parenting [16], family is a major domain not only to reduce child problems but also to enhance positive development even in adverse circumstances. In any situation, and particularly in a disaster context, children depend on their caregivers to understand, process and deal with threats, as well as to get support and guidance. During the confinement situation, with an uninterrupted contact between child and family, family interactions and parenting behaviors emerge as clear candidates to be examined as potential sources of risk and protection.

### 1.2. Parenting in COVID-19 Times

Previous research has shown that, in adverse situations, the way that parents try to manage a child’s behavior may impact the child’s well-being. It has been shown that, for instance, coercive patterns and harsh parenting predict unfavorable outcomes in children living in stressful situations [17,18]. Similarly, anxious and overprotective styles predicted higher levels of child maladjustment in the context of nature disasters [19,20]. In contrast, the ability to accept, monitor and be positively involved with the child, as well as to encourage problem-solving attitudes, promotes resilience in children subjected to traumatic situations [17].

Overall, parenting practices which usually promote well-being in children are beneficial when children are under difficult circumstances. Nevertheless, beyond such general parenting practices, less is known about the specific parenting practices displayed in relation to the stressful situation, i.e., the way that parents try to ease or support a child’s adaptation to adverse situations, including disasters (see, for example, [21]). In one of the few studies conducted in this regard, Wilson et al. [22] examined parental responses displayed to manage the adaptation to the 9/11 attacks, like media restriction or the way of talking to children about the tragic events. It was found that parent-perceived helpfulness when talking about the attacks predicted less post-traumatic problems in the offspring. Some other studies have also shown that the parents’ willingness to focus on the disaster-related facts and emotions may promote positive adaptations in children [23], whilst avoidant attitudes may be deleterious [19]. Moreover, keeping routines and structure in the family environment seems to exert a positive effect, as routines foster a sense of coherence and manageability [21]. The effects of structured parenting seem to be particularly positive when structure is provided in a non-punitive manner; otherwise, monitoring, structure and control may even result in negative outcomes, as implied by research on the effects of Hurricane Katrina [18,24].

In health-related disasters, like the COVID-19 pandemic, social and health agencies tend to offer some guidelines for how parents should interact with children [25]. For instance, setting home routines, talking to children about the pandemic, controlling media exposure, and involving children in family activities were recommended as effective strategies [26]. However, is still unknown to what extent parents follow these recommendations or whether parenting strategies displayed under these circumstances have effects, positive or negative, on child behavior and overall adjustment.

#### Parenting in a Wider Context of Family Distress

It is important to note that parenting practices are not displayed in a social or family vacuum. Repeatedly, previous research has shown that children’s well-being in adverse situations is affected by the distress levels experienced by parents [27,28], leading researchers to suggest that parenting behaviors might be one of the channels for this effect [29,30,31].

The COVID-19 crisis has subjected families to several health, economic and labor stressors that abruptly disrupted their daily life and routines. Some parents had to keep working outside the home, dealing with the threat of possible contagion, whilst many others kept working from home while dealing with caregiving and homeschooling, without extra-familial support. In other cases, families suffered an economic impact because of unemployment and the collapse of economic markets. In addition, close contagion, hospitalization, taking care of elders and the uncertainty increased fear and turmoil in daily life. As Prime et al. [5] suggested, the social disruption derived from the pandemic and, more specifically, the confinement situation, may affect parents’ well-being which, in turn, will permeate the whole family processes. In fact, according to family systems theory [32,33], it may be expected that the stress affecting one family member will lead to disruptions in the family system and subsystems, including the parent–child dyadic relations. Along this line, another element that has been closely related with parenting practices is the distress experienced, particularly in the role of parents, i.e., when parents feel overwhelmed due to the demands of caregiving and raising children. Considering the COVID-19 times, this parenting distress could have specific triggers, like space restrictions at home, difficulties in keeping children safe and busy and, overall, difficulties in meeting children’s needs and demands whilst coping with their own emotional problems. Prior research suggested that parenting distress could mediate the relationship between parents’ psychological problems and dysfunctional parenting practices [34,35], which, in turn, may also affect child behavior.

Finally, it is important to note that not all parents have the same personal resources to cope with stressful situations. A plethora of constructs have been suggested to cover those variables that protect from life stressors, for instance, hardiness [36], self-efficacy [37], optimism [38] or, more generally, dispositional resilience [39,40]. As in other negative situations, one could expect parents’ internal assets to thrive with changes and difficulties that may affect distress experience during the confinement and, therefore, hold a significant place in the chain of influences on a child’s well-being.

### 1.3. The Present Study

This study was conducted in Spain, one of the first countries affected by the rapid spread of the pandemic, and one of the countries with a higher rate of confirmed cases and deaths per million inhabitants, as of 23 September 2020 (https://www.statista.com/statistics/1104709/coronavirus-deaths-worldwide-per-million-inhabitants/). With the rapid spread of confirmed cases from the beginning of March, the Spanish health system was severely strained, and a strict and prolonged lockdown was enforced. The lockdown started on 14 March 2020, and the population was confined at home, except to purchase first-need products, work or attend emergencies. On 29 March, even more restrictive measures were imposed; all non-essential activity was banned for a period of 14 days, and the job/financial situation of large groups of the population was impacted. The interruption of business operations caused the loss of about one million jobs, and a vast number of contracts were temporarily suspended during the confinement [41]. Additionally, Spain was one of the few countries to force children to stay indoors for weeks, with schools being closed from mid-March. After 43 days, at the end of April, children were allowed to go out on short walks, under very restrictive conditions.

Considering the unique characteristics surrounding the COVID-19 crisis and, more specifically, the confinement situation, the present study aimed to examine, through parent reports, the effects of the extreme Spanish lockdown in children’s behavioral and emotional adjustment, focusing on both negative and positive outcomes and accounting for potential differences based on age. It was also aimed at examining how children’s adjustment was affected by parent-related variables derived from the confinement situation, encompassing relevant aspects that may affect parent–child interactions, i.e., dispositional resilience, emotional response to the COVID-19 crisis and the adaptation of parenting practices to the confinement demands and characteristics. In the absence of longitudinal data, we model children’s behaviors as outcomes of the whole process; nevertheless, this does not preclude the existence of reciprocal effects, as suggested by contemporary research on family relations [42], which emphasizes transactional interplays between the individual and the family context [43]. Based on previous research on parenting, distress and disasters, we aimed to integrate a variety of key aspects about family and child adjustment in a conceptual model, hypothesizing a series of direct and indirect effects of parents’ variables on children’s behavior.

It was firstly expected that, during the confinement, children would experience an increase in behavioral and emotional problems. Nevertheless, as observed in some previous studies [12,13], it was also expected that the confinement situation may also increase specific behaviors that would be indicative of a positive adjustment, including new routine maintenance, a more prosocial involvement and social-oriented reflection, and new efforts to strengthen social bonds. Secondly, higher rates of behavioral problems, including conduct problems and hyperactive behaviors, would be expected for younger children, whilst more elaborate positive behaviors (e.g., social understanding, refinement in beliefs about society) were expected for older children. Thirdly, we hypothesized that the confinement effects on children could be partially explained by parents’ adjustment to the crisis. Hence, we expected that parents’ dispositional resources would affect their personal distress under the confinement circumstances, which, in turn, would trigger an emotional response involving anxiety and depression symptoms. As was observed in previous research [34], parents’ emotional problems derived from the confinement would feed the feeling of burden in the interaction with children (i.e., parenting distress), which would influence specific parenting practices. Finally, as the last step, parenting practices would directly affect children’s behavioral and emotional adjustment to the confinement situation.

## 2. Materials and Methods

### 2.1. Participants

Data for the present study were collected within the Confinement Effects on Families and Children (CONFIA-20) study. This study was conducted in April 2020, during the most restrictive period of the lockdown established by the Spanish Government. It was aimed at evaluating the psychological, emotional and behavioral effects of the confinement in children and families. Data were provided by 1005 (89.5%) mothers, 111 (9.8%) fathers and 7 (0.7%) other parental figures (e.g., grandparents who are in charge of the child) from Galicia (94.2%), NW Spain, with 5.8% participants living in other Spanish regions. Participants provided information on 1123 children (50% girls) aged 3 to 12 years (M_age_ = 7.26; SD = 2.39). As for the parent’s academic level, 61.9% of mothers and 39.6% of fathers, respectively, completed higher education, 22.8% and 28.5% completed vocational training, 8.0% and 11% finished high school and 7.0% and 19.5% completed compulsory education. Only 0.3% of mothers and 1.2% of fathers informed about no academic achievement. At the time of data collection, families spent a mean of 30.87 (SD = 6.37) days confined at home, with around four people (M = 3.90, SD = 1.01) per home. From the parents who were working before the crisis (86.9%), 17.9% kept attending their job, 33.9% were working at home, 19.1% temporarily halted their job, 8.6% were on medical leave and 2.4% lost their job due to the COVID-19 crisis. Finally, families also informed about cases of contagion in a close setting (16.7%), as well as close COVID-related deaths (5.3%).

For the purpose of the current study, we selected data from 940 (89.6%) mothers, 102 (9.7%) fathers and 7 (0.7%) other parental figures, for whom complete baseline data (i.e., no missing values) were available for the main study variables. Participants provided information on 1049 children (50.4% girls; M_age_ = 7.29; SD = 2.39).

### 2.2. Measurements

Given the unique characteristics of the COVID-19 pandemic crisis, and more specifically the confinement situation, some scales were created ad hoc to capture context-related constructs. Further information is given in the description of each new measure, with all item descriptions and factor loadings presented as supplemental material (available online).

#### 2.2.1. Parent-Related Variables

Resilience. The 10-item version of the Connor–Davidson Resilience Scale (CD-RISC-10) [40,44] was used to measure parental dispositional resilience (α = 0.90; MIC = 0.65). Items such as “I am able to adapt when changes occur”, “Having to cope with stress can make me stronger” or “I try to see their humorous side when I am faced with problems” were rated by parents on a four-point Likert scale ranging from 0 (“not at all”) to 4 (“true nearly all the time”).

Perceived distress. Context-related distress, derived from the COVID-19 crisis, was assessed with a four-item scale developed ad hoc for the purposes of the current study. It focused on how the COVID-19 crisis impacted parents’ perception of personal stress, the family economic situation, relations within the family and fear about the future (for further details see Table S1, available online). Parents rated each item, based on their perception, according to what extent the confinement situation affected different aspects of their life (e.g., “Damages the relationships within the family”), on a four-point response scale ranging from 0 (not at all) to 3 (very much). Preliminary analyses showed that the four items significantly correlated with each other (*p* ˂ 0.001) and showed the same pattern of correlations with all study variables. Therefore, for the present study, a composite score encompassing all four items was created (α = 0.66; MIC = 0.44).

Emotional problems. Parents’ emotional problems were assessed with the four-item version of the Patient Health Questionnaire for Depression and Anxiety (PHQ-4) [45], an ultra-brief screening tool to assess anxiety (e.g., “Feeling nervous, anxious, or on edge”; α = 0.73; MIC = 0.58) and depressive symptoms (e.g., “Feeling down, depressed, or hopeless; α = 0.56; MIC = 0.39). To evaluate how the confinement emotionally affected them, parents rated each item on a five-point comparative scale ranging from 0 (much less) to 4 (much more).

Parenting distress. A newly developed measure was used to assess parenting-related distress in the confinement situation; i.e., distress derived by a new context where parents have to deal with regular parenting, academic task supervision, free time management and context-related stressors that may affect child adjustment. Four items were developed ad hoc for the present study (e.g., “Sometimes I have difficulties finding varied activities to get him/her involved”; “It is difficult to give him/her all the attention he/she demands”), which were grouped in a global composite score (α = 0.77; MIC = 0.56) (see Table S2, available online). Parents rated each item on a five-point scale ranging from 0 (strongly disagree) to 4 (strongly agree).

Specific parenting. Specific parenting practices during the confinement were assessed with 15 items created to address the behaviors displayed by parents to help children deal with the COVID-19 crisis. Exploratory factor analysis (EFA) suggested a four-factor solution that was supported by confirmatory factor analysis (CFA) (χ^2^(83) = 272.16; Root mean square error of approximation [RMSEA] = 0.06; Comparative fit index [CFI] = 0.91; Standardized root mean square residual [SRMR] = 0.06) (see Table S3, available online). The first factor defines focused parenting, i.e., the efforts to make the child well informed on the pandemic, and to keep free-flowing communication with him/her on the specific issues of COVID-19 (four items; e.g., “I get into the knowledge he/she has about COVID-19”, “I try to clarify all his/her doubts about the pandemic and its consequences” α = 0.75; MIC = 0.55). A second factor describes a soothing attitude by parents, in an attempt to emotionally comfort the child and keep him/her reassured (three items; e.g., “If needed, I explain that he/she and our family and close friends are safe”, “I frequently show him/her affection: saying I love you, holding him/her; α = 0.54; MIC = 0.35). A third factor refers to structured parenting, i.e., the attempts to give structure and regularity to the child’s daily life (five items; e.g., “I set a daily schedule and an activities plan”, “I try to get him/her to exercise every day”; α = 0.76; MIC = 0.53). A fourth factor comprises items indicating avoidant parenting behaviors, like trying not to talk about COVID-19, and even concealing the seriousness of the situation (three items; e.g., “I avoid talking to him/her about COVID-related issues”, “I try not to let him/her know the seriousness of the problem”; α = 0.66; MIC = 0.48). Items were rated on a five-point scale ranging from 0 (strongly disagree) to 4 (strongly agree).

Socioeconomic status (SES) of parents. SES was indexed through a set of questions about (1) parental level of education and (2) family economic level. Level of education was based on the average of the father’s and mother’s educational level rated on a six-point scale ranging from 1 (without basic studies) to 6 (postgraduate; e.g., PhD). Family economic level was based on parents’ reports of family income rated on a four-point scale from 1 (serious problems in making ends meet) to 4 (well off). A composite SES was computed by first transforming the aforementioned variables into z-scores.

#### 2.2.2. Child Outcomes

Child negative outcomes. To assess negative consequences in child behavior, we used 14 items from the parent-reported Strengths and Difficulties Questionnaire (SDQ) [46], addressing conduct problems (e.g., “Often loses temper”; α = 0.82; MIC = 0.64), emotional problems (e.g., “Often unhappy, depressed or tearful”; α = 0.77; MIC = 0.55) and hyperactive behaviors (e.g., “Restless, overreactive, cannot stay still for long”; α = 0.79; MIC = 0.57). Each scale consists of five items except the conduct problems scale, from which we removed one item (i.e., “Steals from home, school or elsewhere”) because it was not considered informative in the confinement situation. In order to evaluate how the confinement affected child behavior, parents rated each item as compared to child behavior before the lockdown, using a five-point comparative scale of 0 (much less), 1 (somewhat less), 2 (no change), 3 (somewhat more) and 4 (much more). In addition to the mean scores computed for each subscale, categorical variables indicating stability or change in behavior were created based on the comparative response scale. Thus, using scores around 2 as an indicator of stability in behavior, we used a ± 0.5 cut-off over scores equal to 2 to identify groups of children who showed a reduction in the examined behavior (i.e., decrease; average scores ranging from 0 to 1.5), children who did not show any change in behavior (i.e., stable; average scores ranging from 1.5 to 2.5) and children who showed an increase in the examined outcome (i.e., increase; average scores ranging from 2.5 to 4).

Child positive outcomes. In order to evaluate how the confinement positively affected child behavior, a scale of 14 items was created ad hoc for the purposes of the current study. Exploratory factor analysis suggested a four-factor solution that was supported by confirmatory factor analysis (χ^2^(70) = 236.20; RMSEA = 0.07; CFI = 0.91; SRMR = 0.05) (see Table S4, available online). The first factor addressed the child adaptation to daily routines, like school tasks or other planned activities (routine maintenance; four items; e.g., “He/she adapts him/herself to a schedule and daily activity routine”, “Gets involved in schoolwork”; α = 0.55; MIC = 0.34). The second factor denotes the involvement in prosocial activities (prosocial involvement; five items; e.g., “Shows interest to spare time with family”; “Is interested in helping with household chores”; α = 0.70; MIC = 0.46). A third factor addresses changes in socially oriented conceptions and attitudes (social-oriented reflections; three items; e.g., “Acknowledges the value of health workers, as well as other professionals who work to take care of us”, “He/she assumes that we all should collaborate to solve social problems”; α = 0.84; MIC = 0.70). The fourth factor describes the willingness to keep in contact with significant others (social bonding; two items; e.g., “Keeps contact with his/her beloved ones who are not close, by phone, internet…”; α = 0.48; MIC = 0.35). Parents rated each item on the aforementioned five-point comparative scale ranging from 0 (much less) to 4 (much more). Additionally, as described for child negative outcomes, in addition to the mean scores we created, categorical variables were used, describing stability and change in behavior using the ± 0.5 cut-off over scores equal to 2 (i.e., no change), to identify decrease (˂1.5), stable (1.5 to 2.5) and increase (˃2.5) groups from the analyzed outcomes.

### 2.3. Procedure

This study was conducted in the context of a large research line, aimed at studying child behavioral, emotional and social development. The protocols were in accordance with the Declaration of Helsinki and were approved by the Bioethics Committee at the Universidade de Santiago de Compostela. Data for the present study were collected online, from 8 to 27 April, via a secure web-based platform. Participants were recruited through advertisements on the research group’s official webpage, which were also sent to schools, parent associations and social media; the link to the questionnaire was thereafter spread through a snowball technique. To provide informed consent, which implies explicitly agreeing to participate in the study, participants were first informed about the aims and requirements of the study (e.g., anonymous data collection, aimed at families with children aged 3 to 12 years). Participation was anonymous and voluntary. Families were asked to refer their answers strictly to the COVID-19 lockdown situation, and think about one of their children, in case they have more than one. On average, participants took 15 min to complete the questionnaire. There were no reward or compensation for participation.

### 2.4. Statistical Analyses

First, preliminary analyses were performed to examine psychometric properties of the measures developed ad hoc to address the main objectives of the current study. For parenting practices and child positive outcomes, we split up the sample and performed EFA in subsample 1 (*n* = 562; 50.6% girls; M_age_ = 7.34, SD = 2.45) and CFA in subsample 2 (*n* = 561; 49.3% girls; M_age_ = 7.18, SD = 2.32). Additionally, we checked for internal consistency through Cronbach’s alpha and mean inter-item correlation (MIC). The main results from these analyses are described in the Measurements section, with tables provided as supplemental material (available online).

Second, Pearson product-moment correlations were calculated to display the bivariate relationship among the study variables. Third, the effects of confinement on child behavior were examined through prevalence rates of change (i.e., reduction, stable and increase) in child negative and positive outcomes. Given the wide age range, prevalence rates were also examined across age groups, with crosstabs and chi-square tests to examine differences across groups. To this end, three age groups were created including preschool children, aged three to six years (age group 1; *n* = 448; 42.7%), and two groups of school-aged children, one including children aged seven to nine years (age group 2; *n* = 365; 34.8%) and one including children aged 10 to 12 years (age group 3; *n* = 236; 22.5%).

**Figure 1:**
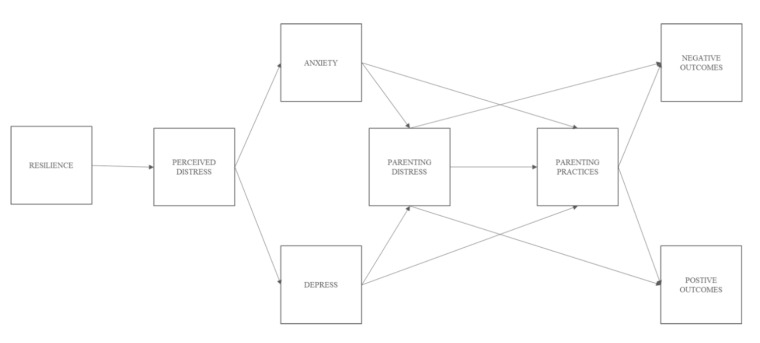
Conceptual path model of associations between parent-related variables and child positive and negative outcomes.

Fourth, the effect of family-related variables (i.e., parents’ resilience, perceived distress, emotional problems, parenting distress and parenting practices), on child negative and positive consequences were modeled by means of path analysis, which permits the simultaneous modeling of several regression relationships. Path analysis was selected because it allows for examining complex models including the direct and indirect (mediated) effects across a set of observed variables. Thus, the mediating role of (1) parenting distress and (2) parenting practices was also examined (see Figure 1). Child’s age and gender, family SES and COVID-related stressors (i.e., close contagion, close COVID-related deaths and job-related variables: keep attending job, working at home) were included as covariates. The model was estimated by the maximum likelihood (ML) method, and goodness-of-fit was assessed using the chi-squared (χ^2^) value, and the RMSEA, CFI and SRMR fit indices. According to suggestions by Hu and Bentler [47], RMSEA and SRMR values lower or equal to 0.06 and 0.05, respectively, and CFI values of 0.95 or higher, are considered indicators of good model fit, whereas an RMSEA and SRMR smaller than 0.08, and a CFI larger than 0.90, indicate adequate model fit. Finally, to test whether path models were moderated by gender and/or age, two sets of multi-group models using (1) gender and (2) the three age groups were computed following Little’s [48] statistical guidelines. Hence, a path model that constrained all the paths to be invariant across groups was compared to a model with paths freely estimated, with the use of the chi-squared (χ^2^) test. Descriptive statistics, correlation analyses and crosstabs were performed using the software package SPSS 20 (IBM, Armonk, NY, USA), whilst EFA, CFA and path analyses were performed in Mplus 7.4 (Muthén & Muthén, Los Angeles, CA, USA) [49].

## 3. Results

### 3.1. Descriptive Information and Correlations between Main Study Variables

Descriptive statistics and correlations between main study variables are presented in Table 1. Among child negative and positive outcomes, the highest mean scores were observed for hyperactive behaviors and social-oriented reflection, respectively. Child’s age was negatively related with parenting distress, soothing, structured and avoidant parenting and with conduct problems and hyperactive behaviors, whilst the correlation was positive with social-oriented reflections and social bonding. Family’s SES was negatively related with perceived distress, avoidant parenting and hyperactivity. Correlations were positive with resilience, soothing, structured parenting and with prosocial behavior. Parent-related variables were overall related with child consequences. In general, whereas parents’ distress and emotional problems were more specifically correlated with child negative outcomes and routine maintenance, specific parenting practices (i.e., focused, soothing, structured and avoidant parenting) were more specifically correlated with child positive outcomes.

### 3.2. Examining Change in Child’s Behavior Derived from the Confinement Situation

The results from Table 2 suggest that a majority of children did not show any change in behaviors addressing negative outcomes (i.e., conduct problems, emotional problems and hyperactive behavior). Nevertheless, some patterns of change were also observed; in this regard, a higher proportion of children increased rather than decreased negative outcomes, particularly for hyperactivity. Regarding child positive outcomes, most children did not show changes in routine maintenance, with only a minor proportion showing a reduction or an increase in this outcome. For prosocial involvement and social bonding, similar proportions of children were distributed across the stable and the increase groups, whilst there was a marked tendency to increase for social-oriented reflection.

Similar prevalence rates were observed across age groups, particularly for emotional problems, routine maintenance and prosocial involvement. There were significant differences across age groups in conduct problems, hyperactivity, social-oriented reflections and social bonding. In this regard, preschool children (i.e., aged 3 to 6 years) showed a higher increase in conduct problems and hyperactivity as compared to their school-aged counterparts. Older children (i.e., aged 10 to 12 years) showed the lowest increase in hyperactivity. There were no differences between seven- to nine-year-old and 10- to 12-year-old children in social-oriented reflections and social bonding, with both groups showing a higher increase as compared to preschool children.

### 3.3. Testing the Effects of Parent-Related Variables on Child Behavior

In order to examine how the confinement affected families and, in turn, child behavior, all study variables were modeled in path analysis, as displayed in Figure 1. Because child negative and positive outcomes were, with some exceptions, overall correlated, the initial model was tested including all study variables, showing an acceptable to good model fit (χ^2^(121) = 462.25, RMSEA = 0.05; CFI = 0.93; SRMR = 0.05). Then, with the aim of examining specific effects on child negative and positive outcomes, two independent models were performed to test parent-related variables on child negative outcomes (path model A) and child positive outcomes (path Model B). Results from the general model basically replicate the results from path models A and B, which are described below (further details for the general model are available upon request). For all analyzed models, we controlled for child’s age, gender, family SES, and COVID-related stressors including close contagion, close COVID-related deaths and job-related variables (i.e., keep attending job, working at home).

#### 3.3.1. Path Model A: Testing Direct Effects of Parent-Related Variables on Child Negative Outcomes

**Figure 2:**
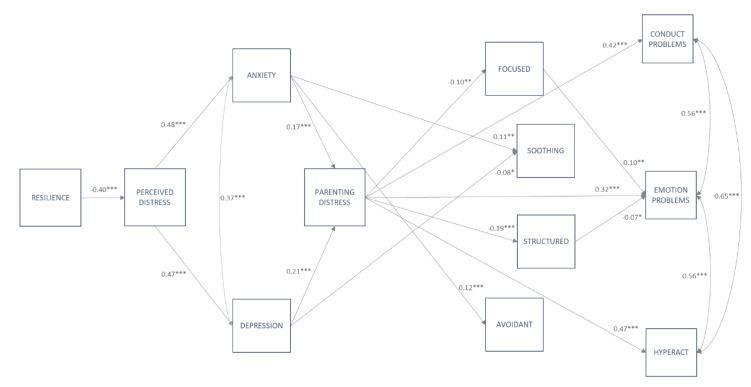
Path model A, including parent-related variables and child negative outcomes. Note: only significant standardized regression coefficients are displayed in the path model. All four parenting practices are inter-correlated; for a clearer presentation of the model, these paths are not displayed. * *p* ˂ 0.05, ** *p* ˂ 0.01 *** *p* ˂ 0.001.

The path model for child negative outcomes acceptably fit the data, χ^2^(67) = 364.99, RMSEA = 0.07, CFI = 0.92, SRMR = 0.06. As displayed in Figure 2, parents’ dispositional resilience exerted a negative effect on perceived distress which, in turn, positively affected parents’ anxiety and depression. Both anxiety and depression were directly and positively related with parenting distress. Additionally, anxiety symptoms were positively related with soothing and avoidant parenting practices, whilst symptoms of depression showed a negative effect on soothing parenting. Parenting distress was negatively related with focused and structured parenting practices, but directly and positively affected child conduct problems, emotional problems and hyperactivity. Finally, with regard to parenting practices and child negative outcomes, only two paths emerged as significant. Focused parenting exerted a positive effect on emotional problems, whilst structured parenting negatively affected child emotional problems.

Accounting for covariates, the results showed that, in regard to parent-related variables, child’s age showed a negative effect on structured (ß = −0.11, *p* ˂ 0.001), soothing (ß = −0.08, *p* ˂ 0.05) and avoidant parenting (ß = −0.20, *p* ˂ 0.001). Family SES exerted a negative effect on perceived distress (ß = −0.11, *p* ˂ 0.001), which was positive on soothing (ß = 0.08, *p* ˂ 0.01) and structured parenting (ß = 0.15, *p* ˂ 0.001). The presence of close contagion (ß = 0.06, *p* ˂ 0.05), going to work (ß = 0.08, *p* ˂ 0.05) and working at home (ß = 0.11, *p* ˂ 0.01) were all positively related with parenting distress. Going to work was also positively related with perceived stress (ß = 0.07, *p* ˂ 0.05) and anxiety (ß = 0.07, *p* ˂ 0.05). Finally, child’s conduct problems and hyperactivity were negatively affected by age (ß = −0.10, *p* ˂ 0.01 and ß = −0.09, *p* ˂ 0.05), whereas only hyperactivity was affected by family SES (ß = −0.11, *p* ˂ 0.001).

In order to further address the potential moderation by age, a multi-group path analysis was also performed. In the first stage, we ran a model that freely estimated the paths separately within each age group, χ^2^(189) = 489.28, RMSEA = 0.07, CFI = 0.92, SRMR = 0.06. As compared to the constrained model, χ^2^(343) = 681.48, RMSEA = 0.05, CFI = 0.91, SRMR = 0.07, the free model fit the data better, suggesting cross-group differences in the paths Δχ^2^(154) = 192.2, *p* ˂ 0.05. Differences in free and constrained models were accounted by three paths, all of them involving associations with one of the covariates included in the model (i.e., SES). Thus, parenting distress was negatively associated with SES only for children aged 10 to 12 years (ß = −0.20, *p* ˂ 0.001); emotional problems was affected by SES only in children aged seven to nine years (ß = 0.09, *p* ˂ 0.05), whereas hyperactivity was negatively affected by SES in preschool children (ß = −0.13, *p* ˂ 0.001) and children aged seven to nine years (ß = −0.12, *p* ˂ 0.001). A final model was checked with these three paths freely estimated across groups, showing an acceptable model fit: χ^2^(337) = 659.83, RMSEA = 0.05, CFI = 0.91, SRMR = 0.07 (further details are available upon request). Potential moderation by gender was also examined, with no significant differences between the free, χ^2^(126) = 424.15, RMSEA = 0.07, CFI = 0.92, SRMR = 0.06, and the constrained model, χ^2^(203) = 500.87, RMSEA = 0.05, CFI = 0.92, SRMR = 0.07, suggesting that path model A was invariant for boys and girls, Δχ^2^(77) = 77.72, *p* ˃ 0.05.

#### 3.3.2. Path Model B: Testing Direct Effects of Parent-Related Variables on Child Positive Outcomes

**Figure 3:**
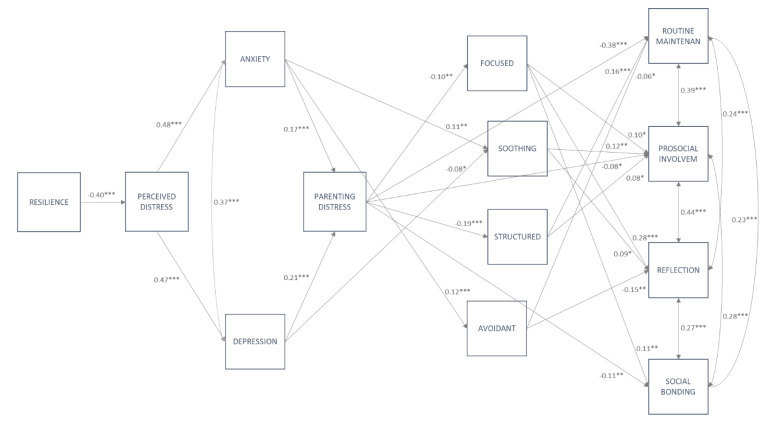
Path model B, including family-related variables and child positive outcomes. Note: only significant standardized regression coefficients are displayed in the path model. All four parenting practices are inter-correlated; for a clearer presentation of the model, these paths are not displayed. * *p* ˂ 0.05, ** *p* ˂ 0.01 *** *p* ˂ 0.001.

The path model of child positive outcomes showed an acceptable to good fit, χ^2^(76) = 316.74, RMSEA = 0.06, CFI = 0.92, SRMR = 0.04. As can be observed in Figure 3, the associations between parents’ resilience, perceived distress, emotional problems, parenting distress and parenting practices were equal to those observed in path model A (Figure 2). In regard to child positive consequences, routine maintenance was negatively affected by parenting distress and, to a lesser extent, by avoidant parenting and (positively) by structured parenting. Prosocial involvement was negatively affected by parenting distress and positively by focused, soothing and structured parenting. Social-oriented reflection was positively affected by focused parenting and negatively by avoidant parenting, with a minor positive association with soothing. Finally, social bonding was negatively affected by parenting distress but positively affected by focused parenting.

Accounting for covariates showed the same results as those observed for path model A. In regard to child positive consequences, child’s age was positively related with social-oriented reflection (ß = 0.20, *p* ˂ 0.001) and social bonding (ß = 0.13, *p* ˂ 0.001), whilst child’s sex was positively related with prosocial involvement (ß = 0.07, *p* ˂ 0.05) and social bonding (ß = 0.08, *p* ˂ 0.05).

The multi-group path analyses, testing for a potential moderation by age, showed that the unconstrained model (χ^2^(216) = 432.39, RMSEA = 0.06, CFI = 0.93, SRMR = 0.05) better fit the data as compared to the constrained model (χ^2^(384) = 639.41, RMSEA = 0.05, CFI = 0.92, SRMR = 0.06), suggesting differences across age groups, Δχ^2^(168) = 207.02, *p* ˂ 0.01. Differences across groups were accounted by five paths. Thus, parenting distress affected soothing and child prosocial involvement only for older children (aged 10 to 12 years; ß = −0.12, *p* ˂ 0.05 and ß = −0.18, *p* ˂ 0.001, respectively). Focused parenting affected social bonding for seven- to nine-year-old and 10- to 12-year-old children (ß = 0.19, *p* ˂ 0.001 and ß = 0.14, *p* ˂ 0.05, respectively) but not for preschoolers. Finally, both parenting distress and social bonding were affected by SES only for older children (aged 10 to 12 years; ß = −0.20, *p* ˂ 0.001 and ß = 0.16, *p* ˂ 0.01). A model with these five paths freely estimated across groups was finally tested, showing an acceptable to good model fit, χ^2^(374) = 600.57, RMSEA = 0.04, CFI = 0.93, SRMR = 0.06 (details available upon request). When testing the potential moderation effect by gender, the results revealed no significant differences between the free, χ^2^(144) = 403.82, RMSEA = 0.06, CFI = 0.92, SRMR = 0.05, and the constrained model, χ^2^(228) = 479.37, RMSEA = 0.05, CFI = 0.92, SRMR = 0.06, suggesting that path model B was invariant across gender groups, Δχ^2^(84) = 75.55, *p* ˃ 0.05.

#### 3.3.3. Testing for Indirect Effects of Parenting Distress and Specific Parenting Practices

The model presented in Figure 1 allowed us to test the potential mediation effects of (1) parenting distress on the relationship between emotional problems and specific parenting practices and (2) specific parenting practices on the relationship between parenting distress and children’s negative and positive outcomes. As displayed in Table 3, testing for potential mediation effects of parenting distress showed three indirect effects that, although small, were still significant and held for both path model A and path model B. Thus, the results showed an indirect effect of anxiety on structured parenting, which was totally mediated by parenting distress. Similarly, parenting distress totally mediated the effect of depressive symptoms on both focused and structured parenting. Unique mediation effects emerged for path model B on the relation between parenting distress and child positive outcomes via focused and structured parenting. As observed in Table 3, focused parenting totally mediated the effect of parenting distress on child social-oriented reflection, whilst structured parenting partially mediated the effect of parenting distress on routine maintenance.

## 4. Discussion

The COVID-19 outbreak forced the imposition of drastic measures to guarantee social distancing all over the world. In Spain, a restrictive lockdown was enacted, which abruptly changed routines and interactions of the population. The present study aimed to examine the psychological effects of the confinement in children and their families, trying to disentangle a pattern of risk and protective factors by examining associations between parent-related variables, derived from the crisis, and child adjustment. As stated in the Introduction, two-way effects are likely to exist between the parenting atmosphere and children’s reactions [43]; within this context, this study was specifically interested in the theory-driven effects of parenting variables on children’s responses to the COVID-19 crisis.

It is noteworthy that the current results revealed that most children (i.e., more than 55%) did not show a relevant change in problematic behaviors during the confinement. Nevertheless, it should also be noted that there were around 30–40% of children who displayed more behavioral disturbances (i.e., conduct problems, emotional problems, hyperactivity) as compared to the pre-confinement situation, raising the importance of addressing the specific effects of confinement in children [7]. Although with some differences in prevalence rates, these results are in line with some previous studies that showed higher levels of anxiety and depressive symptoms during confinement in Chinese children [8], as well as important changes in children’s behavioral and emotional adjustment during the Spanish confinement [9]. As observed in the aforementioned Spanish study, the highest increase was observed for hyperactive behaviors, showing more restlessness, boredom and difficulties in keeping focused [9]. Interestingly, the results also revealed a pattern of positive adjustment, suggesting that the confinement could also raise some positive changes in children. Hence, a higher proportion of children did not show relevant changes in routine maintenance, which reveals a positive routine adaptation despite the straining characteristics of the new situation. In addition, prosocial involvement and social bonding ranged from no change to an increase, whilst social-oriented reflection showed an interesting increase during the confinement. Therefore, even in adverse situations, positive outcomes and personal growth can also be observed [10,11], highlighting the possibilities of psychological gains even when examined at early developmental stages [50].

The results also revealed some significant differences in children’s outcomes based on age. Overall, younger (i.e., preschool) children would be at increased risk of displaying more behavioral problems, including conduct problems and hyperactivity, whilst older children would be more reflective over social-oriented situations. Developmental variables and maturity gaps could explain these differences. Thereby, from a developmental perspective, conduct problems and hyperactive behaviors tend to decrease across childhood [51]. In addition, younger children would have less cognitive, emotional and behavioral resources to process the adverse situation [52], whereas older children would have more resources to adapt to the situation and to learn about the world, as well as to ascribe a new meaning to their experiences [53]. Finally, there were also differences across age groups in social bonding, suggesting that older children were more able to keep contact with other relatives and peers. This could be explained by the direct access to electronic devices that allow online contact with other people (e.g., smart phones, computers), with preschool children being more dependent from their parents or older siblings to maintain online contact with other relatives and, in particular, with their school peers. Importantly, family SES also seems to play an important role in the psychological adjustment in stressful life situations [54], affecting not only child outcomes (i.e., hyperactivity) but also parents’ adjustment and behaviors. As observed in the present study, low SES was related to higher perceived distress, whilst high SES levels were directly related to higher scores in resilience, as well as more soothing and structured parenting.

Therefore, to better understand how the confinement affected children, it is also imperative to explore the effects on the whole family system, including parent–child interactions [5]. During the most restrictive period of the confinement, families had to rapidly adapt their routines, interactions and daily life activities, coping with different health-, work- and family-related sources of stress. In this context, one could expect that, beyond the direct effects of the confinement on children, family-related variables would be likely to constitute risk or protective factors for children. Based on this, one of the main contributions of this study was to link child outcomes to parents’ adjustment and distress variables, all of them integrated in a conceptual framework that models the cascading effects [5] of the COVID-19 crisis on families and children. This approach converges with prior research suggesting that, within crisis and post-disaster situations, children’s psychological well-being would be impacted, at least in part, via the impact on parents, parent–child interactions, specific parenting practices and family environment [19]. This hypothesis was supported in the present study, with results overall showing that the confinement caused an emotional impact on parents that, through direct and indirect effects, affected regular interactions between parents and children (i.e., parenting practices), leading to some relevant changes in child behavior.

More specifically, results showed that the relationship between parenting behaviors and child’s outcomes is complex and not always based on direct effects. In this regard, when predicting both negative (i.e., path model A) and positive outcomes (i.e., path model B), the results clearly showed a more complex pattern of interrelations between parents’ internal assets, distress responses and emotional adjustment. Within this sequence of influences, parents’ dispositional resilience exerted a direct and negative effect on parents’ perceived distress derived from the COVID-19 crisis [40]. Thus, the internal resources brought to the COVID-19 situation (i.e., sense of personal competence, tolerance for adversity, acceptance for changes) seem to protect parents from psychological harms, in line with the previous literature on risk and adaptation to adversities [55], and it might play a relevant role in the sequence of processes which impact the children’s outcomes. The distress experienced by parents, in turn, triggered an emotional response characterized by higher levels of anxiety and depression symptoms. Of note, both perceived distress and anxiety were also affected by parents attending their job, which may place them in a high-risk situation for contagion.

In this regard, the confinement situation led parents to deal with many different stressors and threats (e.g., keeping safe, dealing with job-related issues), including difficulties and challenges in their interactions with their kids (e.g., dealing with child’s school work, meeting child’s needs and demands of attention whilst coping with their own emotional problems). This may create a feeling of discomfort with the caregiving demands (i.e., parenting distress), which, in our model, is shown to be fueled by anxiety and depression symptoms, in line with previous findings in diverse areas of research [35,56].

The role of parenting distress in the analyzed models was noteworthy, since it directly affected parenting practices and child negative and positive outcomes. More specifically, higher levels of parenting distress were significantly related with lower focused and structured parenting, and higher avoidant parenting. The relevance of parenting distress in the prediction of COVID-specific parenting mirrors the results in the field of general parenting, which have shown the relation of parenting distress to an array of dysfunctional parenting behaviors [31]. In the specific context of the COVID-19 crisis, negative emotions associated to the parenthood tasks also seem to prevent parents from using effective parenting practices (e.g., structured), and they may trigger poorer parenting patterns (e.g., avoidant). In addition to these direct effects, parenting distress also mediated the association between parents’ emotional problems and parenting. Hence, via parenting distress, which totally mediated the effects, higher levels of anxiety exerted a negative influence on structured parenting, whilst higher levels of depression negatively influence focused and structured parenting. The mediating role of parenting distress has already been proposed in the literature on general parenting [34,35]; also along this line, previous research has shown that negative perceptions of parenting are connected with emotional problems in adults [57]. It is noteworthy that parenting distress was also affected by COVID-related stressors, including close contagion, keeping up with jobs and working at home. Parenting distress emerges as a compelling variable influenced by both different stressors and emotional impairments that exert great influence on parent–child interactions and, therefore, on child behavior. None of these near stressors was directly related with parenting practices, raising the possibility of parenting distress again mediating these associations. Nonetheless, some effects running from emotional problems (anxiety, depression) toward parenting practices were not mediated by parenting distress. Thus, anxiety was found to positively predict soothing and avoidant parenting. Such results may imply that anxious parents are more willing to protect children from their own anxiety and from other psychological threats, by providing affection and reassurance. This pattern, combined with avoidant behaviors, might be linked to the overprotective style which has previously been found in anxious parents after disasters [20]. Contrariwise, depressive symptoms showed a direct negative effect on soothing behaviors, which may reflect the disengaged attitude of depressive parents in relation to the emotional needs of their children [58].

Focusing on child negative outcomes, parenting distress was revealed as an important risk factor, which exerted stronger influence than parenting practices on child conduct problems, emotional problems and hyperactivity [59,60]. It may be that in an emotionally challenging situations like the COVID-19 crisis, problem behaviors are more responsive to feelings brought by parents to the parent–child relation than to the specific, deliberate strategies to guide children’s behavior; this finding shows the need to promote a good emotional atmosphere at home as a main way to prevent problems in pandemic conditions.

Only emotional problems were directly affected by parenting practices, namely focused and structured parenting. Unexpectedly, focused parenting was positively related with emotional problems. Previous research has shown that both too little and too much talking about disasters may be deleterious for children’s well-being [19]. It might be that the focused parenting, strongly oriented to communication about the pandemic, has the capacity of causing worry and rumination, especially in sensitive children. Additionally, bi-directional influences might be suggested, with anxious children demanding more information, and/or parents trying to support distressed children by offering information [19]. In this regard, it is important to note again that the cross-sectional design developed for the current study did not allow for accounting for potential reciprocal effects between study variables, including the expected influence of child’s behavior on parenting distress [60] and parenting behavior [61], an issue that should be further addressed in future research.

For children’s positive outcomes, both parenting distress and parenting practices acted as relevant factors in predicting child adjustment within a crisis [62]. The results showed that parenting distress negatively affects routine maintenance and, to a lesser extent, prosocial involvement and social bonding, although just for older children. Additionally, the results supported the role of parenting behaviors displayed to deal with the COVID-19 situation (specific parenting practices) as important factors to maintain children’s positive adjustment [22]. Thus, focused parenting influenced prosocial involvement and social-oriented reflection in the whole sample, and social bonding just for older children; soothing triggered more prosocial involvement and social-oriented reflection, and structured parenting favored structured routine maintenance and higher prosocial involvement. It is not surprising that parenting behaviors more linked to the informational and emotional dimensions of the pandemic (i.e., focused, soothing) exert their influence on the cognitive (i.e., social reflection) and socio-affective outcomes (i.e., prosocial involvement, social bonding); meanwhile, the efforts of parents to bring regularities to their children’s lives may be successful for the establishment of healthy habits (i.e., routine maintenance). Regarding avoidant parenting, it negatively affected prosocial involvement and social-oriented reflection; the literature on trauma has demonstrated that avoidant behaviors by parents deprive children of opportunities to understand the situation, correct misconceptions and make sense of the adverse experiences [22]; in a similar way, avoidance of contents and emotions related to the pandemic seems to hinder the emergence of positive outcomes in children. Interestingly, focused and structured parenting also exerted indirect effects on children’s positive outcomes, mediating the association between parenting distress and social-oriented reflection and routine maintenance, respectively. In general, our results suggest that parenting distress may act as an underlying canvas for both the negative and the positive reactions to the pandemic; meanwhile, context-related parenting practices seem not to be so relevant to avoiding behavioral problems, but they do have a significant role as levers, which may push towards positive growth during the COVID-19 times.

### 4.1. Theoretical and Practical Implications

The psychological effects of the COVID-19 outbreak are still emerging and beginning to be known [1,2]. Among them, the psychological effects of the crisis, social distancing and social isolation measures (i.e., restrictive lockdowns) on children and their families constitute a great challenge in the field [3,7]. The current results revealed that most children did not show important changes in behavior, although there were noticeable increasing rates in conduct and emotional problems, and hyperactivity. Interestingly, the current findings also raised the possibility that during the confinement, children developed a positive pattern of adaptation, increasing prosocial behaviors, with better routines of activities and self-care, and developed more mature conceptions about society and health [13].

Following a theoretically driven approach, these results also supported a cascade of effects from parents’ emotional responses to children’s positive and negative adjustment, via parenting distress and parenting practices [27,28,30]. Therefore, the importance of parent-related variables to examine and better understand children’s adaptation and well-being in the context of a crisis was overall supported [5]. Among them, parenting distress emerged as a key factor, driving important effects on parenting practices and child’s behavior, which particularly affected child negative outcomes. The results also highlight the importance of examining specific parenting practices, displayed in exceptional circumstances, such as the confinement, to ease children’s distress and promote their well-being [21]. Although much more research is needed, the current findings suggest that parenting practices based on setting home routines and structuring daily activities, talking about the pandemic, keeping openness to the crisis-related emotions, showing affection and involving children in family activities act as relevant factors, particularly in promoting children’s positive adjustment. These strategies, among many others, are in line with most of the guidelines and suggestions provided by different health agencies [25] and child-focused NGOs [26].

Based on the foregoing, the current results may help to better inform researchers, social agents and policy makers to develop effective guidelines for future outbreaks with the aim of promoting positive parenting [63] and fostering children’s well-being in the family. To this end, prevention programs specifically targeted to family needs within the context of a health-related crisis are particularly needed. Some scholars have already proposed specific tools to boost well-being in families and children during pandemic times [64,65], which, according to our results, might help to improve family adaptation and parenting behaviors. Because some of the associations are moderated by age, preventive strategies and guidelines should also consider children’s ages to improve the impact of their effects. Additionally, family SES might determine how families face stressful situations, suggesting that prevention and intervention proposals should be sensitive to many different socioeconomic circumstances. Altogether, these suggested strategies will help to promote a positive pattern of adjustment in children and their families.

### 4.2. Strengths and Limitations

This study was conducted in a large sample of Spanish families, during the acute period of the lockdown, allowing for examination of the confinement effects on children and their families. It covered great heterogeneity in child positive and negative outcomes, as well as a wide range of parent-related variables that could better inform future strategies to improve children’s and families’ adjustment to new potential outbreaks and other health-related crises. Of note, some of the analyzed variables were created ad hoc to cover the specific needs of the research on the COVID-19 crisis. Model fit indices showed an adequate fit, with all items showing factor loadings above 0.30, which is considered an appropriate cut-off for factor loadings [66]. In terms of internal consistency, Cronbach’s alpha was modest for some scales (e.g., social bonding), which may compromise their reliability. However, although Cronbach’s alpha is a common measure for internal consistency, several concerns about its reliance have been raised, such as its dependence on the number of the items, or the problems stemming from unrealistic assumptions (e.g., the lack of adherence to tau equivalence, or the normal distribution of the items) [67]. In order to overcome these limitations, the MIC was used as a more informative index of internal consistency than the alpha, with all values being indicative that the developed scales have relatively good internal consistency. In general, the new scales presented in this research are intended to serve as a starting point for the emerging research on COVID-19 adaptations. Nevertheless, further analyses on their psychometric properties are required in order to generalize their use in future research based on a similar health-related crisis. One of the aims of further research should be focused on the age appropriateness of some measures (e.g., positive outcomes), so that the items may be tailored to the developmental specificities of different age groups. In fact, it cannot be disregarded that the differential age-related functioning of some items (e.g., “Gets involved in schoolwork”, “Assumes the importance of health caring”) partially explain the differences found across age groups.

The current findings should also be interpreted in the light of the following limitations. First, although all study variables were modeled assuming a sequential pattern of direct effects, the cross-sectional design did not allow for establishing causal effects and conclusions. Moreover, we have already indicated that that bidirectional effects are likely to occur among parent and child variables [60,61]; hence, longitudinal designs are needed for a better account of the family processes involved in health-related crises. Importantly, the existence of cascading effects from the psychosocial context to the child’s adaptations does not imply that the reversed effects are negligible [5]. So far, the study of bidirectional relations between parents and children in collective disasters has been very scarce; some studies have reported that, within this kind of context, the effects mainly run from parents to children, and not in the opposite direction [68]. Nevertheless, more research is clearly needed in order to delineate the reciprocal influences within the family dynamics in the COVID-19 crisis.

Second, relying on parent reports may have raised the possibility that observed effects were partially due to shared method variance. In addition, because in most cases the informant was the mother, the generalizability of the current results to other parental figures could be modest. Similarly, since the present study was mainly conducted in one region of Spain, its geographic generalizability is limited, requiring new cross-national research in this field. Third, given the conditions of anonymity, we were not able to identify how many families participated in the study and, therefore, in which cases parents provided information on more than one child. Fourth, online data collection allowed for reaching a wide number of families in a timely manner; nevertheless, sampling biases (i.e., participants are mainly parents with easy access to the internet) might have affected the findings; likewise, the lack of direct supervision during the questionnaire administration might have worked against the motivation for careful responses in some parents. Fifth, an arbitrary cut-off was used to identify changes in children’s behavior as compared to the pre-confinement situation. Although it was based on a comparative response scale, it may cause differences across previous studies that used alternative methodological approaches to examine changes in children’s behavior. Additionally, multiple relevant variables have been considered in this study, trying to capture the nuances and specificities of the COVID-19 crisis; nevertheless, a more thorough model of parenting in health-related disasters would involve other less specific factors, like social support or general parenting practices (e.g., warmth, harsh parenting), which have also been involved in children’s responses to adversities [18]. Finally, although current results did not reveal differences by gender in path relations, future research should also account for more specific gender differences across age groups. Similarly, accounting for potential differences based on parents’ gender (i.e., collecting data from both parents on the same child), as well as other relevant family variables (e.g., family structure, family SES, type of house, place of residence), would be relevant to further understand how a health-related crisis impacts family systems, and its effect on children’s psychological adjustment.

## 5. Conclusions

The COVID-19 confinement disturbed the emotional and behavioral patterns of children, but also allowed some positive adaptations to flourish. This study shows that the psychological impact on children is closely linked to the impact on parents. In a widespread health crisis like COVID-19, parents are likely to be affected by the situation, and therefore they are themselves at a high risk for general distress and emotional problems, which result in poorer child outcomes. Parenting distress seems to occupy a central role in the stream of effects from parents’ emotional disturbance to children’s adjustment; parenting distress could transport the COVID-related distress into the interactions with children, and, thus, it could bridge the roles of “parents as persons” (with their worries and frustrations) and “parents as parents” (providers of caregiving and guidance to children) [69]. In practical terms, interventions designed to foster positive adaptations to the COVID-19 crisis should be mainly aimed at: (a) reducing the emotional impact on parents, by strengthening the abilities of parents to cope with the social and personal threats derived from the crisis; (b) boosting parents’ willingness to appropriately address the pandemic-related facts and emotions in their interactions with children; (c) encouraging parents to provide a structured family environment so that healthy habits may be maintained and, at the same time, uncertainty and uncontrollability may be buffered; (d) developing the skills to keep calm, reassure and show affection to children even in challenging times.

## Figures and Tables

**Table 1:** Descriptive statistics and correlations between main study variables.

	1.	2.	3.	4.	5.	6.	7.	8.	9.	10.	11.	12.	13.	14.	15.	16.	17.	18.
1.Age	-																	
2.SES	−0.02	-																
3. Resilience	−0.02	0.14 ***	-															
4. Perceived distress	−0.02	−0.33 ***	−0.41 ***	-														
5. Anxiety	−0.05	−0.03	−0.36 ***	0.48 ***	-													
6. Depression	−0.03	−0.06	−0.34 ***	0.47 ***	0.52 ***	-												
7. Parent distress	−0.20 ***	−0.03	−0.23 ***	0.35 ***	0.29 ***	0.30 ***	-											
8. Focused	0.03	0.05	0.19 ***	−0.07 *	−0.04	−0.07 *	−0.12 ***	-										
9. Soothing	−0.09 **	0.09 **	0.17 ***	−0.03	0.06 *	−0.03	0.01	0.43 ***	-									
10. Structured	−0.08 **	0.16 ***	0.26 ***	−0.15 ***	−0.09 **	−0.12 ***	−0.20 ***	0.52 ***	0.40 ***	-								
11. Avoidant	−0.19 ***	−0.07	−0.09 **	0.10 **	0.13 ***	0.06	0.09 **	−0.17 ***	0.18 ***	0.00	-							
12. Conduct prob.	−0.18 ***	−0.04	−0.20 ***	0.27 ***	0.30 ***	0.28 ***	0.45 ***	−0.07 *	−0.01	−0.12 ***	0.10 **	-						
13. Emotional prob.	−0.04	−0.01	−0.17 ***	0.26 ***	0.31 ***	0.28 ***	0.32 ***	0.04	0.06 *	−0.07 *	0.04	0.62 ***	-	-				
14. Hyperact.	−0.17 ***	−0.13 ***	−0.24 ***	0.31 ***	0.30 ***	0.31 ***	0.48 ***	−0.03	0.01	−0.11 ***	0.08 *	0.73 ***	0.62 ***	-				
15. Routine	0.05	0.07 *	0.23 ***	−0.21 ***	−0.15 ***	−0.17 ***	−0.42 ***	0.16 ***	0.05	0.26 ***	−0.11 ***	−0.33 ***	−0.25 ***	−0.38 ***	-			
16. Prosocial	−0.01	0.07 *	0.23 ***	−0.09 **	−0.03	−0.10 **	−0.11 ***	0.21 ***	0.19 ***	0.20 ***	−0.07 *	−0.19 ***	−0.09 **	−0.11 ***	0.43 ***	-		
17. Reflection	0.23 ***	0.03	0.10 **	0.02	0.05	0.03	−0.05	0.34 ***	0.17 ***	0.15 ***	−0.21 ***	−0.06	−0.07 *	0.02	0.26 ***	0.46 ***	-	
18. Social bonding	0.15 ***	0.05	0.09 **	−0.07 *	−0.01	−0.04	−0.16 ***	0.14 ***	0.08 *	0.09 **	−0.07 *	−0.10 **	−0.02	−0.09 **	0.28 ***	0.31 ***	0.32 ***	-
Mean	7.29	0.02	2.52	1.37	2.64	2.39	2.26	3.35	3.46	3.21	1.71	2.29	2.22	2.43	1.95	2.56	2.78	2.52
SD	2.39	0.75	0.68	0.61	0.71	0.74	0.90	0.52	0.48	0.56	0.84	0.71	0.62	0.60	0.58	0.57	0.65	0.96
Range (Min-Max)	3.00–12.00	−2.63–1.26	0.00–4.00	0.00–3.00	0.00–4.00	0.00–4.00	0.00–4.00	0.00–4.00	0.00–4.00	0.00–4.00	0.00–4.00	0.00–4.00	0.00–4.00	0.2–4.00	0.00–4.00	0.2–4.00	0.67–4.00	0.00–4.00

* *p* ˂ 0.05, ** *p* ˂ 0.01 *** *p* ˂ 0.001.

**Table 2:** Distribution of children in decrease, no change, and increase groups for negative and positive outcomes, in total sample and across age groups.

	Total Sample			Age Group 1 (3–6 years)			Age Group 2 (7–9 years)			Age Group 3 (10–12 years)			
	Decrease	Stable	Increase	Decrease	Stable	Increase	Decrease	Stable	Increase	Decrease	Stable	Increase	χ^2^ (df = 4)
	*n* (%)	*n* (%)	*n* (%)	*n* (%)	*n* (%)	*n* (%)	*n* (%)	*n* (%)	*n* (%)	*n* (%)	*n* (%)	*n* (%)	
Conduct prob.	75 (7.1%)	666 (63.5%)	308 (29.4%)	29 (6.5%)	248 (55.4%)	171 (38.2%)	21 (5.8%)	255 (69.9%)	89 (24.4%)	25 (10.6%)	163 (69.1%)	48 (20.3%)	34.82 ***
Emotional prob.	85 (8.1%)	677 (64.5%)	287 (27.4%)	41 (9.2%)	276 (61.6%)	131 (29.2%)	27 (7.4%)	234 (64.1%)	104 (28.5%)	17 (7.2%)	167 (70.8%)	52 (22.0%)	6.29
Hyperactivity	40 (3.8%)	595 (56.7%)	414 (39.5%)	17 (3.8%)	232 (51.8%)	199 (44.4%)	11 (3.0%)	211 (57.8%)	143 (39.2%)	12 (5.1%)	152 (64.4%)	72 (30.5%)	13.66 **
Routine	150 (14.3%)	787 (75.0%)	112 (10.7%)	66 (14.7%)	335 (74.8%)	47 (10.5%)	63 (17.3%)	267 (73.2%)	35 (9.6%)	21 (8.9%)	185 (78.4%)	30 (12.7%)	8.98
Prosocial	22 (2.1%)	506 (48.2%)	521 (49.7%)	9 (2.0%)	217 (48.8%)	222 (49.6%)	8 (2.2%)	165 (45.2%)	192 (52.6%)	5 (2.1%)	124 (52.5%)	107 (45.3%)	3.16
Reflection	6 (0.6%)	402 (38.3%)	641 (61.1%)	5 (1.1%)	216 (48.2%)	227 (50.7%)	0 (0.0%)	116 (31.8%)	249 (68.2%)	1 (0.4%)	70 (29.7%)	165 (69.9%)	38.64 ***
Social bonding	120 (11.4%)	435 (41.5%)	494 (47.1%)	67 (15.0%)	198 (44.2%)	183 (40.8%)	34 (9.3%)	150 (41.1%)	181 (49.6%)	19 (8.1%)	87 (36.9%)	130 (55.1%)	18.07 **

** *p* ˂ 0.01 *** *p* ˂ 0.001.

**Table 3:** Standardized indirect effects of (1) anxiety and depression on parenting practices through parenting distress, and (2) parenting distress on child outcomes through parenting practices.

	β	95% CI
(1) Path Model A and Path Model B		
ANX— Parenting distress—Structured	−0.03 ***	−0.05, −0.02
DEP—Parenting distress—Focused	−0.02 **	−0.04, −0.01
DEP– Parenting distress—Structured	−0.04 ***	−0.06, −0.02
(2) Path Model B		
Parenting distress—Focused—Reflection	−0.03 **	−0.03, −0.01
Parenting distress—Structured—Routine	−0.03 **	−0.03, −0.01

Only significant standardized indirect effects are presented. ** *p* < 0.01, *** *p* < 0.001.

## Supplementary Materials

The following are available online at https://www.mdpi.com/1660-4601/17/19/6975/s1, Table S1: Items and factor loadings for the perceived distress scale, Table S2: Items and factor loadings for the parenting distress scale, Table S3: Items and factor loadings for the parenting practices subscales, Table S4: Items and factor loadings for the child positive outcomes subscales.
