# Supplementary material for: Testing the Effects of COVID-19 Confinement in Spanish Children: The Role of Parents’ Distress, Emotional Problems and Specific Parenting
Source: Int J Environ Res Public Health. 2020 Sep 24;17(19):6975. doi: 10.3390/ijerph17196975 (PMC7578923; doi:10.3390/ijerph17196975)
Supplement: Supplementary file 1 [file ijerph-17-06975-s001.zip › ijerph-902625-supplementary/S1_Table.pdf]

The current situation…		Perceived distress
1.	Causes you stress	.74
2.	Affects your family's economic situation	.39
3.	Damages the relationships within the family	.62
4.	Raises your fear about the future	.58
