# Supplementary material for: Testing the Effects of COVID-19 Confinement in Spanish Children: The Role of Parents’ Distress, Emotional Problems and Specific Parenting
Source: Int J Environ Res Public Health. 2020 Sep 24;17(19):6975. doi: 10.3390/ijerph17196975 (PMC7578923; doi:10.3390/ijerph17196975)
Supplement: Supplementary file 1 [file ijerph-17-06975-s001.zip › ijerph-902625-supplementary/S2_Table.pdf]

		Parenting distress
1.	I have difficulties to get him/her involved in schoolwork	.61
2.	Sometimes, I have difficulties to find a variety of activities to get him/her involved	.61
3.	It is difficult to find some time for me	.67
4.	It is difficult to give him/her all the attention he/she demands	.80
