# Supplementary material for: Testing the Effects of COVID-19 Confinement in Spanish Children: The Role of Parents’ Distress, Emotional Problems and Specific Parenting
Source: Int J Environ Res Public Health. 2020 Sep 24;17(19):6975. doi: 10.3390/ijerph17196975 (PMC7578923; doi:10.3390/ijerph17196975)
Supplement: Supplementary file 1 [file ijerph-17-06975-s001.zip › ijerph-902625-supplementary/S3_Table.pdf]

	Focused	Soothing	Structured	Avoidant
10. I get into the knowledge he/she has about the covid-19	.51			
12. I show him/her some of the preventive and protective actions we all should take (e.g., washing hands properly)	.67			
13. I try to solve his/her doubts about the covid-19, and its consequences	.78			
14. I encourage him/her to express his/her emotions about the situation	.73			
4. I try to keep calm so my child cannot feel my anxiousness		.46		
5. If needed, I explain that he/she and our family and close friends are safe		.36		
15. I often show him/her affect (saying "I love you", holding him/her)		.46		
6. I set a daily schedule and activities plan			.55	
7. I try to get him/her to exercise every day			.68	
8. Under the current circumstances, I try that he/she keeps a well- balanced diet			.62	
9. I try to share leisure activities with my child			.68	
11. I control the time he/she spends watching TV, and the kind of informative programs he/she watches			.59	
1. I avoid talking to him/her about covid-related issues				.67
2. I try not to let him/her know the seriousness of the problem				.86
3. I tell him/her not to worry				.36
