# Supplementary material for: Testing the Effects of COVID-19 Confinement in Spanish Children: The Role of Parents’ Distress, Emotional Problems and Specific Parenting
Source: Int J Environ Res Public Health. 2020 Sep 24;17(19):6975. doi: 10.3390/ijerph17196975 (PMC7578923; doi:10.3390/ijerph17196975)
Supplement: Supplementary file 1 [file ijerph-17-06975-s001.zip › ijerph-902625-supplementary/S4_Table.pdf]

He/she…	Routine maintenance	Prosocial involvem.	Social-oriented reflection	Social bonding
1. Is creative (draws, does handicrafts…)	.62			
2. Gets involved in schoolwork	.49			
5. Shows interest in his/her hygiene and personal care	.41			
14. Adapts him/herself to a schedule and daily activity routine	.32			
3. Shows interest to spare time with family		.55		
4. Shows interest for supportive social activities (e.g., applaud at 8pm)		.67		
6. Is interested in helping with household chores		.50		
7. Shows affect to others		.43		
8. Is able to appreciate the good things in everyday life, even the simplest		.54		
9. Acknowledges the value of health workers, as well as other professionals who work to take care of us			.77	
10. Assumes that we all should collaborate to solve social problems			.85	
11. Assumes the importance of health caring			.78	
12. Keeps contact with his/her beloved ones who are not close, by phone, internet…				.71
13. Keeps contact with other peers outside the family context (e.g., schoolmates, friends…)				.46
